# Supplementary material for: Chiral magnetic chemical bonds in molecular states of impurities in Weyl semimetals
Source: Sci Rep. 2019 Jun 11;9:8452. doi: 10.1038/s41598-019-44842-8 (PMC6560129; doi:10.1038/s41598-019-44842-8)
Supplement: Supplementary file 2 — Supplementary File S2 [file 41598_2019_44842_MOESM2_ESM.pdf]

Chiral magnetic chemical bonds in molecular states of impurities in Weyl semimetals

Y. Marques[1], W. N. Mizobata[1], R. S. Oliveira[1], M. de Souza[2], M. S. Figueira[3], I. A. Shelykh[4,5], A. C. Seridonio[1,2,*]

[1] *Departamento de Física e Química, Unesp - Univ Estadual Paulista, 15385-000, Ilha Solteira, SP, Brazil*
[2] *IGCE, Unesp - Univ Estadual Paulista, Departamento de Física, 13506-900, Rio Claro, SP, Brazil*
[3] *Instituto de Física, Universidade Federal Fluminense, 24210-340, Niterói, RJ, Brazil*
[4] *Science Institute, University of Iceland, Dunhagi-3, IS-107, Reykjavik, Iceland*
[5] *ITMO University, St. Petersburg 197101, Russia*

**corresponding author: antonio.seridonio@unesp.br*

SUPPLEMENTARY INFORMATION

Analytical derivation of spatial dependent self-energy given by Eq.(10)

Upon deriving the induced local density of states described by the Eq.(9) in the main text, the spatial dependent self-energy given by Eq.(10) emerges as the main quantity defining the impurity scattering process. Its derivation, in which we set $\hbar = 1$, is as follows:

$$\varSigma_{\sigma}^{\chi}(\boldsymbol{r}_{mj}) = \frac{\nu_0^2}{N}\tilde{\mathcal{G}}_{\sigma\chi}^{0}(\varepsilon,\mathbf{r}_{mj}) = \frac{\nu_0^2}{N}\sum_{k}\frac{\varepsilon + i\eta - \sigma\chi\left(k_z - \chi Q_z\right)}{\left(\varepsilon+i\eta\right)^2 - \nu_F^2\left(k-\chi Q\right)^2}e^{-i\boldsymbol{k}\cdot\boldsymbol{r}_{mj}}, \tag{1}$$

where $\tilde{\mathcal{G}}_{\sigma\chi}^{0}(\varepsilon,\mathbf{r}_{mj})$ is the Green's function of the pristine system at $\boldsymbol{r}_{mj}$, which represents the relative position between the impurity site and the position where the LDOS evaluation is performed, ν_0 denotes the impurity-host hybridization amplitude, N is the total number of states and ν_F is the Fermi velocity. For a sake of simplicity in k-space, we introduce the convenient shift $k - \chi Q \to k$ and find

$$\varSigma_{\sigma}^{\chi}(\boldsymbol{r}_{mj}) = \frac{\nu_0^2}{N}e^{-i\chi\boldsymbol{Q}\cdot\boldsymbol{r}_{mj}}\int\frac{d^3k}{\Delta\Omega_k}\frac{\varepsilon+i\eta-\sigma\chi k_z}{\left(\varepsilon+i\eta\right)^2-\nu_F^2k^2}e^{-i\boldsymbol{k}\cdot\boldsymbol{r}_{mj}} = I_1 + I_2, \tag{2}$$

with $\varDelta\Omega_k = \frac{(2\pi)^3}{\Omega}$ being the elementary volume and $N = \frac{\int d^3k}{\varDelta\Omega_k} = \frac{\Omega D^3}{6\pi^2 v_F^3}$.

The first integral I_1 is defined by

$$I_1 = \frac{3\nu_0^2 v_F^3}{4\pi D^3}e^{-i\chi\boldsymbol{Q}\cdot\boldsymbol{r}_{mj}}\int_0^{\infty}\frac{\left(\varepsilon+i\eta\right)k^2}{\left(\varepsilon+i\eta\right)^2-\nu_F^2k^2}\int_0^{\pi}e^{-i|k||\boldsymbol{r}_{mj}|\cos\theta}\sin\theta\int_0^{2\pi}d\phi d\theta dk. \tag{3}$$

Upon carrying out the spherical integration and due to the symmetry of the integrand in k, I_1 can be recast as

$$I_1 = \frac{3\nu_0^2 v_F^3}{2D^3}e^{-i\chi\boldsymbol{Q}\cdot\boldsymbol{r}_{mj}}\int_{-\infty}^{\infty}dk\frac{\left(\varepsilon+i\eta\right)k}{\left(\varepsilon+i\eta\right)^2-\nu_F^2k^2}\frac{e^{-ik|\boldsymbol{r}_{mj}|}}{i|\boldsymbol{r}_{mj}|}. \tag{4}$$

After evaluating a contour integration, we obtain

$$I_1 = -\frac{3\pi\nu_0^2 v_F}{2D^3}e^{-i\chi\boldsymbol{Q}\cdot\boldsymbol{r}_{mj}}\left[\frac{\varepsilon}{|\boldsymbol{r}_{mj}|}e^{-i|\boldsymbol{r}_{mj}|\frac{\varepsilon}{\nu_F}}\right]. \tag{5}$$

For the second integral I_2 defined in Eq.(2), we have

$$I_2 = -\frac{\nu_0^2}{N}e^{-i\chi\boldsymbol{Q}\cdot\boldsymbol{r}_{mj}}\int\frac{\Omega d^3k}{(2\pi)^3}\frac{\sigma\chi k\cos\theta}{\left(\varepsilon+i\eta\right)^2-\nu_F^2k^2}e^{-i\boldsymbol{k}\cdot\boldsymbol{r}_{mj}}. \tag{6}$$

To evaluate I_2, we should follow the same procedure adopted in solving I_1. This yields

$$I_2 = -\frac{3\pi\nu_0^2 v_F}{2D^3}e^{-i\chi\boldsymbol{Q}\cdot\boldsymbol{r}_{mj}}\left(\pm\frac{\sigma\chi\varepsilon}{|\boldsymbol{r}_{mj}|}\pm i\frac{\sigma\chi\nu_F}{|\boldsymbol{r}_{mj}|^2}\right)e^{-i|\boldsymbol{r}_{mj}|\frac{\varepsilon}{\nu_F}}. \tag{7}$$

Therefore, the sum $I_1 + I_2$ gives the Eq.(10) of the main text, where $\pm$ signs correspond to the vector direction, i.e., positive for $\mathbf{r}_{mj}$ and negative for $\mathbf{r}_{jm}$.
